# Air and temperature sensitivity of n-type polymer materials to meet and exceed the standard of N2200

**DOI:** 10.1038/s41598-020-60812-x

**Published:** 2020-03-04

**Authors:** Samantha Brixi, Owen A. Melville, Brendan Mirka, Yinghui He, Arthur D. Hendsbee, Han Meng, Yuning Li, Benoît H. Lessard

**Affiliations:** 1 Department of Chemical and Biological Engineering, University of Ottawa, 161 Louis Pasteur, K1N 6N5 Ottawa, Ontario Canada; 2 Department of Chemical Engineering, University of Waterloo, 200 University Avenue West, Waterloo, ON N2L 3G1 Canada

**Keywords:** Engineering, Materials science, Nanoscience and technology, Physics

## Abstract

N-type organic semiconductors are notoriously unstable in air, requiring the design of new materials that focuses on lowering their LUMO energy levels and enhancing their air stability in organic electronic devices such as organic thin-film transistors (OTFTs). Since the discovery of the notably air stable and high electron mobility polymer poly{[N,N′-bis (2-octyldodecyl)- naphthalene-1,4,5,8-bis(dicarboximide)-2,6-diyl]-alt-5,5′-(2,29-bisthiophene)} (N2200), it has become a popular n-type semiconductor, with numerous materials being designed to mimic its structure. Although N2200 itself is well-studied, many of these comparable materials have not been sufficiently characterized to compare their air stability to N2200. To further the development of air stable and high mobility n-type organic semiconductors, N2200 was studied in organic thin film transistors alongside three N2200-based analogues as well as a recently developed polymer based on a (3E,7E)-3,7-bis(2-oxoindolin-3-ylidene)benzo[1,2-b:4,5-b′]difuran-2,6(3 H,7 H)-dione (IBDF) core. This IBDF polymer has demonstrated promising field-effect mobility and air stability in drop-cast OTFTs. While N2200 outperformed its analogues, the IBDF-based polymer displayed superior air and temperature stability compared to N2200. Overall, polymers with more heteroatoms displayed greater air stability. These findings will support the development of new air-stable materials, and further demonstrate the persistent need for the development of novel n-type semiconductors.

## Introduction

Semiconducting polymers may hold the key to the future of scalable high-production volume flexible electronics, with their solution processable properties making them transferrable to roll-to-roll printing technologies. Newly developed materials must exhibit high charge mobilities and air stability to be viable for commercial success, and numerous recent materials have achieved these desired characteristics^1–6^. Exceptional performance of the charge transporting semiconductor layer is essential to the fabrication of effective devices. Performance must be sustained under a variety of environmental stressors that would be experienced under typical fabrication and operation conditions, including exposure to air or elevated temperature. Particularly, it is desirable for a material to be shelf-stable under ambient conditions. Electron-transporting n-type materials are especially susceptible to air-induced degradation under ambient conditions^7^. Recent developments in the design of air-stable n-type materials has led to the discovery of the material poly{[N,N9-bis(2-octyldodecyl)-naphthalene-1,4,5,8-bis(dicarboximide)-2,6-diyl]-alt-5,59-(2,29-bisthiophene)} (N2200), which was found to have exceptional air-stability among comparable materials in a protected top-gate configuration, along with excellent electron mobility^8^. In the hopes of achieving and improving upon the highly desirable characteristics of N2200, numerous materials have been developed based on this NDI core^2,9–12^. While polymers of this class have received significant focus, newer classes of polymers have also been developed with promising results. One such example are IBDF-based polymers (also referred to as BDOPV), which have shown promising charge transport characteristics and air stability^13–18^. However, the air stability of many of these materials is not well characterized, and little to no information is known about their response to temperature.

Charge transport has been studied through theoretical and experimental studies for numerous organic semiconductors. For amorphous and polycrystalline films, which are typical morphologies for thin films of organic semiconductors, an increase in mobility is observed with increasing temperature. However, in particularly high-mobility and highly crystalline materials, band-like transport may be observed, wherein mobility decreases with temperature^19^. These studies are almost exclusively performed at temperatures below ambient to accurately determine modes of charge transport^19–22^. While these studies are important for enhancing understanding of charge transport in organic semiconductors, it is important to ensure that these findings hold true under conditions relevant to practical operation. This is particularly important for polymers due to the dependence of their morphology on temperature, which can have a significant impact on charge transport. Studies reporting material stability rarely include a side-by-side comparison of results to other well-known materials as a reference point, despite the ambient conditions (such as relative humidity) being significant factors in material stability, which differ in each laboratory these materials are studied. Our study attempts to control for these factors by comparing relative stability of all materials in the same time frame to remove this confounding factor.

**Figure 1:**
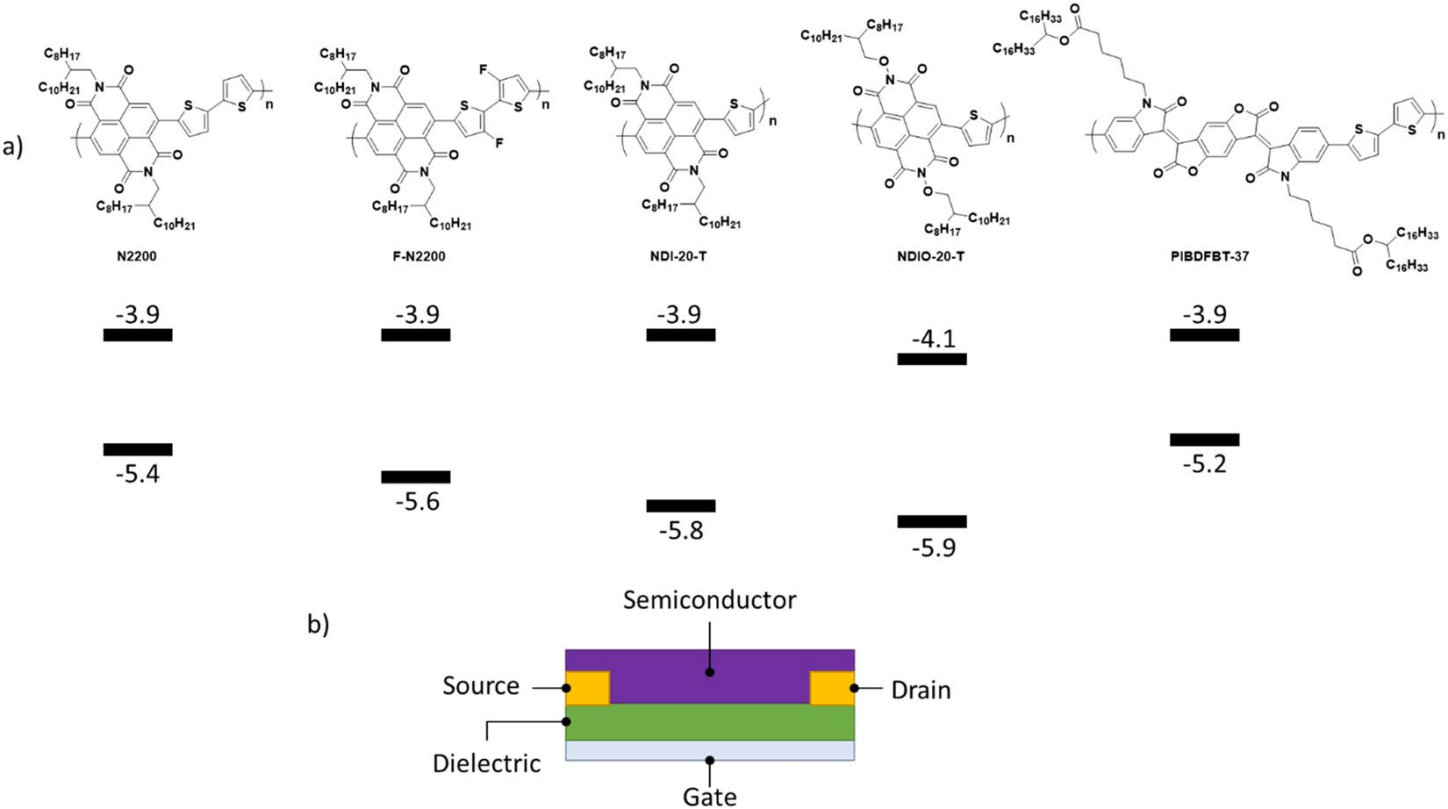
(**a**) Polymers studied in this report and their respective frontier energy levels. (**b**) Structure of a bottom gate bottom contact organic thin film transistor (OTFT). Corresponding lowest unoccupied molecular orbital (LUMO) and highest occupied molecular orbital (HOMO) energy levels for each polymer obtained through cyclic voltammetry (CV) and UV-visible spectra presented in Fig. S1.

This study examines the materials N2200, a fluorinated derivative (FN2200), a derivative lacking a thiophene (NDI-20-T), an alkoxy derivative (NDIO-20-T), and another material based on a (3E,7E)-3,7-bis(2-oxoindolin-3-ylidene)benzo[1,2-b:4,5-b′]difuran-2,6(3H,7H)-dione core (IBDF) PIBDFBT-37 or simply PIBDF. Figure 1 compares the polymer structures as well as the reported lowest unoccupied molecular orbital (LUMO) and highest occupied molecular orbital (HOMO) energy levels. Towards the goal of implementing practical applications for these high performing n-type polymer materials, their response to the effect of air and temperature were studied. This information will assist in informing future materials design choices for organic electronics and will be useful towards the design of new molecules to have desired properties such as air stability.

## Results and Discussion

### The influence of air

To investigate the effects of environment on the polymer semiconductors, organic thin film transistors (OTFTs) were fabricated with each material shown in Fig. 1 as the active semiconductor. Devices were fabricated by drop casting in the bottom-gate bottom-contact (BGBC) configuration to ensure the materials were adequately exposed to the environment. To characterize their electrical properties, the OTFTs were first characterized under vacuum (P < 0.1 Pa) followed by characterization in air. The resulting electrical properties are summarized in Table 1. The N2200 used in this study was prepared by two different methods; first, a sample prepared by Stille coupling (provided by 1-Material), and a second sample prepared by direct heteroarylation (provided by Brilliant Matters Organic Electronics). Both yielded identical results, thus the values for the 1-Material polymer are presented below.

**Table 1:** Properties of n-type polymers at 30 °C in air or vacuum environment.

	N2200		F-N2200		NDI-20-T		NDIO-20-T		PIBDFBT-37	
	Vacuum	Air	Vacuum	Air	Vacuum	Air	Vacuum	Air	Vacuum	Air
µ_*e*_ (cm^2^/Vs)	0.06	0.03	0.03	0.005	0.006	0.002	0.001	0.0006	0.1	0.02
I_on/off_	10^3^	10^2^	10^4^	10^3^	10^4^	10^3^	10^3^	10^3^	10^4^	10^3^
V_T_ (V)	23.9	33.9	−0.31	10.6	11.4	18.4	15.5	32.9	32.1	41.5
HOMO (eV)^a)^	−5.4		−5.6		−5.8		−5.9		−5.2 (−5.7)^b)^	
LUMO (eV)^a)^	−3.9		−3.9		−3.9		−4.1		−3.9	

^a)^Lowest unoccupied molecular orbital (LUMO) and highest occupied molecular orbital (HOMO) energy levels (eV) were obtained by CV and band-gaps determined by UV-vis, presented in Fig. S1.

^b)^Values presented in brackets were obtained directly from the onset of oxidation in CV experiments, while all other values are calculated by combining the band-gap from UV-visible spectroscopic studies with the LUMO value obtained by CV.

The highest electron mobilities (*µ*_*e*_) were observed for N2200 and PIBDF with values of 0.06 and 0.1 cm^2^/Vs, respectively. F-N2200 also displayed relatively high mobility at 0.03 cm^2^/Vs. Although the *µ*_*e*_ obtained for N2200 has been reported above 1 cm^2^/Vs in top-gate configurations^23^, the values obtained in this study correspond with values found for unoptimized devices on Si/SiO_2_ in bottom gate, bottom contact (BGBC) OTFT configuration^24^. The performance of these materials corresponds to values in literature^8,12,13,25^. Minor differences can be attributed to differences in device architecture and processing method, as these material have previously been characterized with spin-coated devices.

Typically for n-type polymers, a deeper LUMO is less susceptible to charge trapping, thus requiring a lower bias to inject charge, resulting in a lower observed *V*_*T*_^26^. In this case, there is little difference in the frontier energy levels of each of these polymers, yet the *V*_*T*_ range from 0 to 30 V. This implies that the difference in *V*_*T*_ cannot be entirely accounted for by this traditional explanation. The performance of numerous materials^27–30^, including N2200^8^, have been shown to vary with film morphology. Therefore, it is possible the difference in *V*_*T*_ between each polymer observed under vacuum is not due to relative LUMO energies, but instead the film morphology contributes to this difference. This variation in device performance with morphology is often attributed to charge traps formed at grain boundaries^31,32^, which can significantly affect *V*_*T*_^33^. The presence of charge traps at grain boundaries also has the added effect of decreasing the mobility, as a result of the mobile charges becoming localized to these trap states^32^. This may be a further explanation for the differences in observed *µ*_*e*_ compared to literature values, due to a different morphology obtained by drop-casting rather than the typical spin-coating.

The presence of air shifts the *V*_*T*_ in the positive direction and decreases *µ*_*e*_, indicating p-type doping or electron trapping of the material by air. This is consistent with previous work with p-type polymers^34^, n- and p-type small molecules^35,36^ and ambipolar carbon nanotubes^37^. It is known that electron traps formed at the semiconductor-dielectric interface require a more positive gate voltage to become filled and allow electron transport, thereby increasing *V*_*T*_^38^. It is likely these traps act as p-type dopants and account for the observed effect for both p- and n-type polymers. This phenomenon is mainly observed in SiO_2_ dielectrics^39^. The occurrence of interface traps can be observed in the shape of the subthreshold region in a transfer curve (representative transfer curves can be found in Fig. S2), and calculation of the device subthreshold swing (*S*_*w*_) allows for the number of interface traps (*N*_*it*_) to be estimated^40^. The *S*_*w*_ and *N*_*it*_ have been estimated for each of these devices in vacuum and in air, shown in Table 2. For all materials, a greater *N*_*it*_ was found in air, confirming that air exposure increases number of interface traps.

**Table 2:** Subthreshold swing (*S*_*w*_) characteristics and number of interface traps (*N*_*it*_*)* of devices characterized in vacuum and in air at room temperature.

	*S*_*w*_ (V/dec)		*N*_*it*_ (cm^−2^ V^−1^)	
	Vacuum	Air	Vacuum	Air
N2200	4.7	5.1	7.4 × 10^12^	8.0 × 10^12^
FN2200	4.1	5.5	6.4 × 10^12^	8.7 × 10^12^
NDI-20-T	2.9	3.1	4.6 × 10^12^	4.9 × 10^12^
NDIO-20-T	3.7	5.5	5.7 × 10^12^	8.7 × 10^12^
PIBDFBT-37	6.4	6.9	1.0 × 10^13^	1.1 × 10^13^

The mechanism of the interaction of oxygen and water in air with N2200 has been studied rigorously to determine the individual effects by water and oxygen by Di Pietro *et al*.^41^. The loss of mobility in air is reportedly due to an interaction between oxygen molecules and the fused benzene core of the naphthalene diimide unit. Each of the other NDI-containing polymers in this study could exhibit these same interactions. Similarly, the IBDF-based polymer contains fused π-conjugated rings at its core, and oxygen may exhibit similar interactions. Water was found to have the combined effect of creating electron traps as well as contributing to the degradation of the polymer film. This would explain why all materials see the same decrease in mobility in air by an order of magnitude under the combined effects of water and oxygen. It is interesting to note that N2200 had the least decrease in mobility compared to the other four polymers. Based on the results of Di Pietro’s work, this could indicate that oxygen has a greater electronic interaction with the other polymers.

The effect of electron trapping is also observed in the decrease of the on/off ratio. Each material saw a decrease in *I*_*on/off*_ of approximately one order of magnitude, with the exception of NDIO-20-T. The differences in on/off ratio were due to the decrease of the on current in air, however the off current decreased as well in all materials to a smaller degree.

### The influence of temperature in vacuum (*P* < 0.1 Pa)

**Figure 2:**
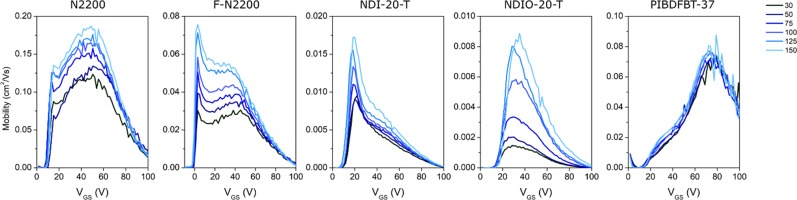
Effect of temperature under vacuum *(P* < *0*.*1* *Pa)* on electron mobility vs gate voltage (*V*_*GS*_) of various n-type semiconducting polymers all in a bottom gate bottom contact (BGBC) organic thin film transistor (OTFT) device configuration.

The performance of these polymers at elevated temperature was also evaluated, giving insight into the mechanism of charge transport. The prepared devices were characterized under vacuum from 30 °C to 150 °C and the corresponding electron mobility over the gate voltage (*V*_*GS*_) range can be found in the *µ*_*e*_ curves presented in Fig. 2, and corresponding transfer curves may be found in Fig. S3. In the field of organic electronics, there is a push for more accurate and responsible representation of material mobility and device performance^42–44^, and as such, the figures presented examine the mobility of the materials over the given *V*_*GS*_ range. The characteristic shape of these *µ*_*e*_ curves is due to contact resistance, which is reported to have two significant effects: (1) the overestimation of *µ*_*e*_ at low *V*_*GS*_ resulting in a peak in the *µ*_*e*_ curve^45^, and (2) a decrease in the measured mobility at high *V*_*GS*_, giving an overall downward trend of the *µ*_*e*_ curve^46^. These features result in what has been described as the electrical double slope^44^, which is the observation of two distinct slopes, or regions of linearity in the saturation curves. These features are observed in the presented *µ*_*e*_ curves, and by examining changes across the entire measured *V*_*GS*_ range, the changes in mobility can be accurately compared.

The mobility curve for all polymers increased with increasing temperature, but the degree of the effect varied significantly between polymers (Fig. 2). While PIBDF saw only a 30% increase in mobility at its maximum mobility, the mobility of NDIO-20-T increased by nearly 500%. The *V*_*T*_ of each material, which can be inferred from the *µ*_*e*_*-V*_*GS*_ curves by the sharp increase in *µ*_*e*_ of each curve, did not substantially change with temperature. All materials exhibit the electrical double-sloping that results in a downwards trend of mobility at high *V*_*GS*_, however only F-N2200 and NDI-20-T exhibit the sharp peak in *µ*_*e*_ that is likely due to the contact resistance between the materials and electrodes.

The lack of change in *V*_*T*_ for these materials as temperature rises suggests that the density of electron charge traps is not changing, which could imply there is little change in the morphology or water content of the films as a result of changing temperature. These devices had all previously been exposed to air, so it is reasonable to assume that oxygen and water were adsorbed onto the films and dielectric interface, as was discussed previously. The devices were subjected to a vacuum atmosphere for 1 hour prior to testing to ensure any oxygen and water were desorbed. Due to the lack of significant shift in *V*_*T*_ in the tested temperature range, it appears this was sufficient to remove any oxygen and water that would cause charge trapping. It is also likely there is little to no significant morphological change at the interface, as a change in size or number of grain boundaries would be expected to cause a change in *V*_*T*_^32^. The devices were pre-annealed under vacuum to 200 °C in an attempt to minimize effects of changing morphology, so that the mainly the electronic effect of temperature could be isolated. Therefore, the results observed with respect to the variation of *µ*_*e*_ with temperature can be assumed to be mainly caused by the varying temperature, not due to the loss of charge traps caused by altered morphology or oxygen and/or water content.

The increase in *µ*_*e*_ with increasing temperature agrees with most modern charge transport mechanisms for both amorphous and polycrystalline materials, such as charge hopping or mobility edge, respectively. It has been previously suggested that N2200 exhibits electron hopping^47^. The very small effect of temperature on PIBDF may be an indication of low dependence on charge carrier concentration, as charge carrier concentration is temperature-dependent^20^, and *µ*_*e*_ is well-known to be dependent on charge carrier concentration^48^. This could also be indicative of a morphological change at increased temperature, which may interfere with organization at the dielectric interface, thus negating any positive effect of temperature on *µ*_*e*_ within the bulk of the film^49^. Further study may elucidate the nature of charge transport of the IBDF polymer.

### The influence of temperature with air exposure

**Figure 3:**
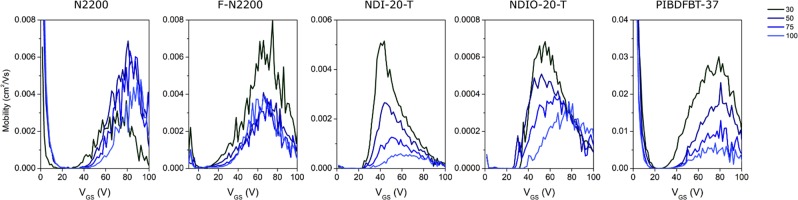
Effect of temperature in air on electron mobility vs gate voltage (*V*_*GS*_) of various n-type semiconducting polymers all in a bottom gate bottom contact (BGBC) organic thin film transistor (OTFT) device configuration.

It is also important to examine the effect of air and temperature simultaneously. The presence of oxygen and moisture in the air may compound with the temperature effects previously observed. The same experiments carried out under vacuum were also performed in air, increasing the temperature at intervals from 30 °C to 100 °C, maintaining this temperature for 30 minutes prior to testing, and the corresponding electron mobility at varied *V*_*GS*_ can be found in Fig. 3.

The trends observed in vacuum are reversed when devices are heated while exposed to air. In the presence of air, the performance is decreased with increasing temperature. PIBDF is among the most strongly affected by this, despite being the least affected by temperature under vacuum. *µ*_*e*_ is decreased at high temperature for all materials, and *V*_*T*_ is increased as well. The increased *V*_*T*_ could indicate a difference in morphology or in chemical structure. Corresponding transfer curves can be found in Fig. S4.

**Figure 4:**
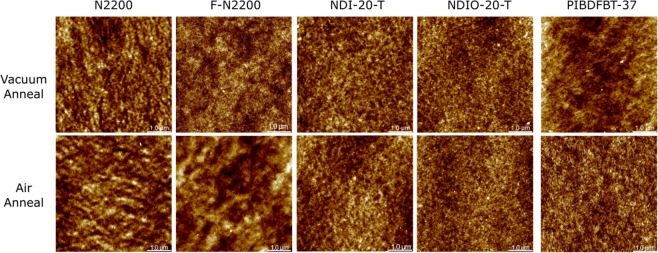
Atomic force microscopy (AFM) of 5 µm × 5 µm sections of each polymer film after annealing in vacuum and air.

As previously noted, charge injection depends on the LUMO of the material. If the material becomes irreversibly oxidized, this change in structure will change the frontier energy levels and could potentially lead to an increase in the *V*_*T*_ as observed here. Morphological changes caused solely by the increasing temperature are unlikely, as it was observed that no *V*_*T*_ shift occurred under vacuum. However, a change in chemical structure may also induce morphological change, particularly at higher temperature. From Table 3, it is clear that heating samples in the presence of air negatively affects the morphology, as all materials show increased roughness except for PIBDF, which showed a decreased roughness as determined using atomic force microscopy (Fig. 4).

**Table 3:** Surface RMS roughness (nm) of drop-cast polymer samples following to heating in either air or vacuum at 100 °C determined using atomic force microscopy.

	N2200	F-N2200	NDI-20-T	NDIO-20-T	PIBDFBT-37
Vacuum heated	1.13	1.23	0.884	0.786	2.16
Air heated	1.23	1.77	1.21	0.988	1.76

**Table 4:** Percent change in characteristic peak height intensity of UV-visible spectra.

	N2200	F-N2200	NDI-20-T	NDIO-20-T	PIBDFBT-37
Peak (nm)	705	685	620	605	790
Air	−18.4%	−22.4%	−25.0%	−11.4%	−0.4%
Vacuum	−4.2%	−14.4%	−16.3%	−19.8%	+0.7%

It has been proposed that due to the long aliphatic chains of N2200, the interaction with the core of the semiconductor at dielectric interface is minimized^8^, and the unaligned polymer will only take on a face-on orientation if annealed above 300 °C^50^. As each of these polymers contain similarly sized aliphatic chains, this could be contributing to the instability at elevated temperatures under ambient conditions, as the core of the polymer does not interact directly with the dielectric, leading to the potential of diffusion of oxygen and moisture to this interface. The different composition and branching point of the chain on the IBDF polymer may contribute to reducing this effect, leading to greater air stability.

### Conserved changes from heating in air

**Figure 5:**
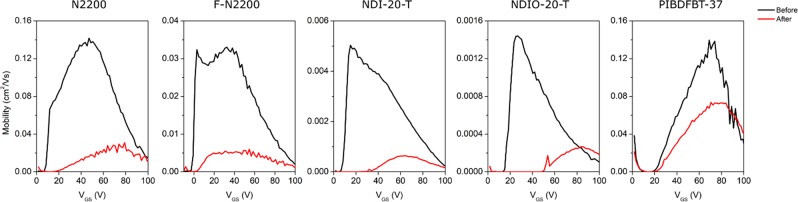
Electron mobility (*µ*_*e*_) vs gate voltage (*V*_*GS*_) of various n-type semiconducting polymers before and after heating films to 100 °C in air. All polymers characterized in a bottom gate bottom contact (BGBC) organic thin film transistor (OTFT) device configuration in a vacuum atmosphere.

To examine whether the observed decreases in *µ*_*e*_ and increases in V_T_ due to heating in air were reversible, the performance of the materials at 30 °C under vacuum was examined prior to and immediately after heating the samples to 100 °C in air. Figure 5 illustrates the difference observed using *µ*_*e*_ vs *V*_*GS*_ plots for each material.

For all NDI-based materials, the positive *V*_*T*_ shift and loss of *µ*_*e*_ was conserved. The mobility of PIBDF-based OTFTs settled at approximately 50% of their original value, where all other materials sustained at least 80% decrease in mobility. These significant losses were not observed in N2200, F-N2200, or NDIO-20-T at elevated temperature (Fig. 3). Yet, PIBDF saw a significant decrease in performance in air at high temperature, but this was not conserved when the device was re-exposed to vacuum. This may indicate that no significant chemical changes occurred to PIBDF when exposed to air at high temperature, while NDI-based materials were more susceptible to permanent damage. The loss of *µ*_*e*_ observed in Fig. 3 for PIBDF could be explained by an enhancement of the interaction with either water or oxygen resulting in a mostly reversible increase in the number of electron charge traps present.

### Long-term stability

**Figure 6:**
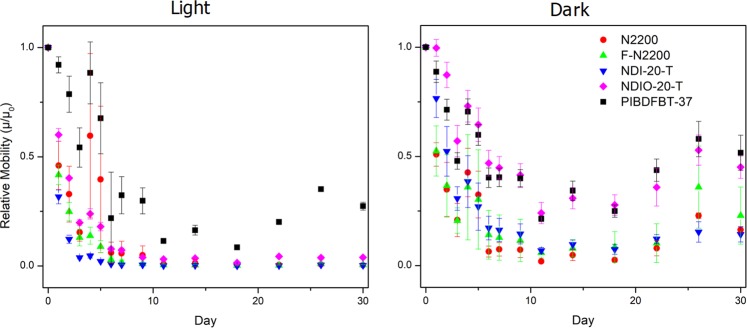
Average of normalized mobilities (µ/µ_day 0_) for various n-type semiconducting polymers characterized daily over a period of one month. Each chip contains four 20 µm channel length devices which were characterized in air and averaged for each data point each day.

Finally, the stability of each material was examined over time (30 days) under ambient conditions in an OTFT, both in the presence of indoor light and in the dark (Fig. 6).

When exposed to light, the *µ*_*e*_ of all devices decreased faster and to a greater extent. Triplet oxygen is expected to be the main culprit in the degradation of N2200^41^. As each of these materials is photoactive, this rapid decrease in *µ*_*e*_ compared to OTFTs stored in the dark could also be explained by electrons being photo-excited to the LUMO, and this excited state would readily react with oxygen. Di Pietro *et al*. also noted that other materials besides N2200 appear to undergo the same degradation, so this mechanism is likely to affect a wide range of materials^41^. However, PIBDF appears to be the most stable material when exposed to light, and matched by NDIO-20-T in the dark. Some fluctuation in results are likely due to variations in relative humidity (RH), but generally these are quite small. Devices were all measured on the same days and at the same time each day to make differences comparable within the dataset.

While all the materials see less decrease in *µ*_*e*_ while stored in the dark, PIBDF maintains the highest performance. Most materials experience over an order of magnitude decrease in *µ*_*e*_ (some even over 3 orders of magnitude when stored in light) except for PIBDF which largely retains its high *µ*_*e*_ throughout the study (Fig. 6). Despite PIBDF having the smallest band gap of the materials, absorbing in the NIR region, it has impressive stability in air under ambient conditions when exposed to light. The loss of *µ*_*e*_ over time could be caused on the short time scale by the formation of charge traps by adsorbed water and oxygen. This is reflected in the similar rate of *µ*_*e*_ decrease in the first 7 days of the study. Beyond this, the continued performance loss in the materials stored in light may be caused by slow chemical degradation by the UV- and visible-light activation of electrons in the semiconductors that are then reacted with oxygen and water in the air to degrade the film. NDIO-20-T appears to be the most strongly affected by the presence of light compared to the other materials. This may be due to the relative strength of the N-C and N-O bonds of the side chains, with bond energies of 305 kJ/mol and 201 kJ/mol, respectively. The weaker N-O bond may be more susceptible to cleavage in the presence of light.

### UV-Visible spectroscopy

**Figure 7:**
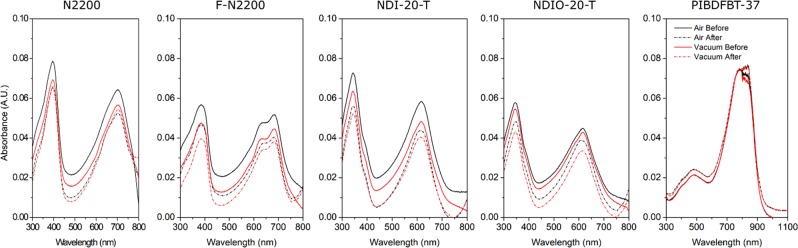
Normalized UV-vis comparison of thin films of n-type polymer materials before (solid lines) and after (dotted lines) heating to 130 °C in air (black) or vacuum (red).

To further examine the effect of air on the material, UV-Visible spectroscopy was used to determine whether chemical changes occurred in the material. Degradation of a particular absorbance peak indicates the loss of a particular chromophore, while the growth of a new peak indicates the formation of a new chromophore and overall absorbance will decrease if the degree of conjugation is decreased^51–54^. Glass slides were spin-coated with the materials and their absorbances were measured. Following this, each slide was heated at 130 °C either in vacuum or in air to simulate the testing observed in the vacuum and air-temperature studies (Fig. 7).

A clear trend emerges from the UV-visible spectra of these materials. For all materials except PIBDF and NDIO-20-T, there is a greater loss of absorbance intensity after exposure to heat in air than in vacuum. In NDIO-20-T, the magnitude of the change is greater following heating in vacuum. The only notable change in PIBDF is the shift of the absorption maximum at 750 nm and 850 nm, where before heating the peak is higher at 790 nm, and after heating higher at 850 nm, which occurs in both air and vacuum. The magnitude of these changes are given in Table 4. This is consistent with the smaller relative loss of *µ*_*e*_ as a result of heating the PIBDF in air, as was shown in Fig. 5. These results suggest the changes in *µ*_*e*_ and *V*_*T*_ observed in the NDI polymer based devices when heated in air, as these differences in the UV-visible spectra may indicate changing chemical structure, and irreversible changes to the material. These spectra further suggest that PIBDF is more stable than the other NDI based polymers.

### Developing structure-property relationships towards more stable polymers

As previously discussed, these materials have similar LUMO levels and therefore it cannot be concluded that the relative stability of the polymers is related to molecular orbital energies in these examples. However, the differing structures of these materials enable a greater understanding of the effect structural changes have on air stability. It has previously been shown that heteroatoms increase chemical stability of semiconductors due to the stabilization of radicals^55^. Further supporting this, heteroatoms such as N, O, and S have been reported to significantly increase stability of radicals^56^. We can conclude that additional heteroatoms appear to lend enhanced stability in the example of the IBDF polymer being highly air-stable compared to the other examples presented in this work. This same factor may also lend greater air-stability to N2200, as it is possible the additional thiophene unit and subsequent incorporation of additional sulfur atom also enable additional radical stabilization, preventing degradation from air exposure. Moreover, N2200 is reported to have an optimized conformation interacting with oxygen that is stable^41^. This conformation involves the twisting of the thiophene units to form a “cradle” around the O_2_ molecule. The addition of fluorine to the thiophene linkers in the form of F-N2200 or the removal of one thiophene linker in NDI-20-T could alter this interaction and lead to a less favourable conformation that would result in a more detrimental interaction of O_2_ and polymer, and therefore lower observed air stability.

## Conclusion

This study examined the environmental sensitivities of four polymers based on an NDI core, and one polymer based on an IBDF core. OTFTs were fabricated with each of these polymers and studied with varied temperature in air and vacuum environments. In vacuum, N2200 was not strongly affected by temperature compared to the similar NDI-based polymers, but PIBDF showed the lowest temperature response overall. In air, all polymers showed decreasing *µ*_*e*_ with increasing temperature, however N2200 was the least affected by elevated temperature in air. To expand on these results, the stability of these materials under ambient conditions was studied over a month-long period. PIBDF showed the highest stability when exposed to light, while both NDIO-20-T and PIBDF were the most stable materials when devices were stored in the dark under ambient conditions. The stability of PIBDF was further supported by the lack of change in its UV-visible spectrum when heated in air, compared to the decrease in overall absorbance of the other materials, indicating that the PIBDF is not chemically altered by exposure to heated air while the other materials are. These studies support previous conclusions about the enhancement of air-stability in n-type materials through the inclusion of heteroatoms and suggest that the unique conformation of the NDI-2Th units in N2200 could lead to its comparative air stability next to other NDI-based polymers. These results are promising for the further development of materials for high-performance n-type air-stable polymers.

## Experimental

### Device preparation

Prior to semiconductor deposition, pre-patterned Si/SiO_2_ substrates with gold source-drain electrodes (W = 2000 µm, L = 20 µm; Fraunhofer IPMS) were first washed with acetone and dried with N_2_, then plasma-treated for 15 minutes to clean. The surfaces of the substrates were functionalized with octyltrichlorosilane (OTS) by heating in a 1% (v/v) solution of OTS in toluene at 70 °C for 1 hour. Substrates were then dried for 1 hour under vacuum at 70 °C to remove residual toluene. Semiconducting polymers were dissolved in chloroform at 1 mg/mL and drop cast with 1 µL drops onto the cleaned substrates on the channels. Finally, devices were annealed at 200 °C for four hours under vacuum. N2200 was obtained from 1-Material and Brilliant Matters Organic Electronics. FN2200 was obtained from 1-Material. NDI-20-T^12^, NDIO-20-T^12^, and PIBDFBT-37^13^ were synthesized according to the literature.

### Electrical characterization

A custom Electrical probes station, oesProbe A10000-P290 (Element Instrumentation Inc. & Kreus Design Inc.) with Keithley 2614B were used to perform electrical measurements under controlled atmosphere. Device performance was measured in the saturation region, with control source-drain voltage (*V*_*DS*_) maintained at a constant 60 V, while gate voltage (*V*_*GS*_) was varied from −10 V to 100 V to obtain measurements of source-drain current (*I*_*DS*_). Equation 1:

1 $${I}_{DS}=\frac{\mu {C}_{i}W}{2L}{({V}_{GS}-{V}_{T})}^{2}$$

where *µ* is the field-effect electron mobility of the material, *C*_*i*_ is the capacitance, *W* is the width of the channel, *L* is the length of the channel, and *V*_*T*_ is the threshold voltage. By taking the square root of Eq. 1, a linear relation is obtained (as shown in Eq. 2), so that the *µ* and *V*_*T*_ can be calculated directly from the slope and x-intercept of an $\sqrt{{I}_{DS}}\,vs\,{V}_{GS}$ curve, respectively.

2 $$\sqrt{{I}_{DS}}=\sqrt{\frac{\mu {C}_{i}W}{2L}}({V}_{GS}-{V}_{T})$$

Finally, the on/off ratio is determined by the ratio of *I*_*on*_ and *I*_*off*,_ which are the highest and lowest currents, respectively, measured in the characterized gate voltage range.

## Supplementary information

Supplementary information
