# Supplementary material for: Air and temperature sensitivity of n-type polymer materials to meet and exceed the standard of N2200
Source: Sci Rep. 2020 Mar 4;10:4014. doi: 10.1038/s41598-020-60812-x (PMC7055259; doi:10.1038/s41598-020-60812-x)
Supplement: Supplementary file 1 — Supplementary information [file 41598_2020_60812_MOESM1_ESM.docx]

**Air and temperature sensitivity of n-type polymer materials to meet and exceed the standard of N2200**

*Samantha Brixi^1^, Owen A. Melville^1^, Brendan T. Mirka^1^, Yinghui He,^2^ Arthur D. Hendsbee,^2^ Han Meng,^2^ Yuning Li^2^ and Benoît H. Lessard^1,*^*

^1^Department of Chemical and Biological Engineering, University of Ottawa, 161 Louis Pasteur, K1N 6N5, Ottawa, Ontario, Canada

^2^Department of Chemical Engineering, University of Waterloo

*correspondence: Benoit.lessard@uottawa.ca

**Supporting Information**


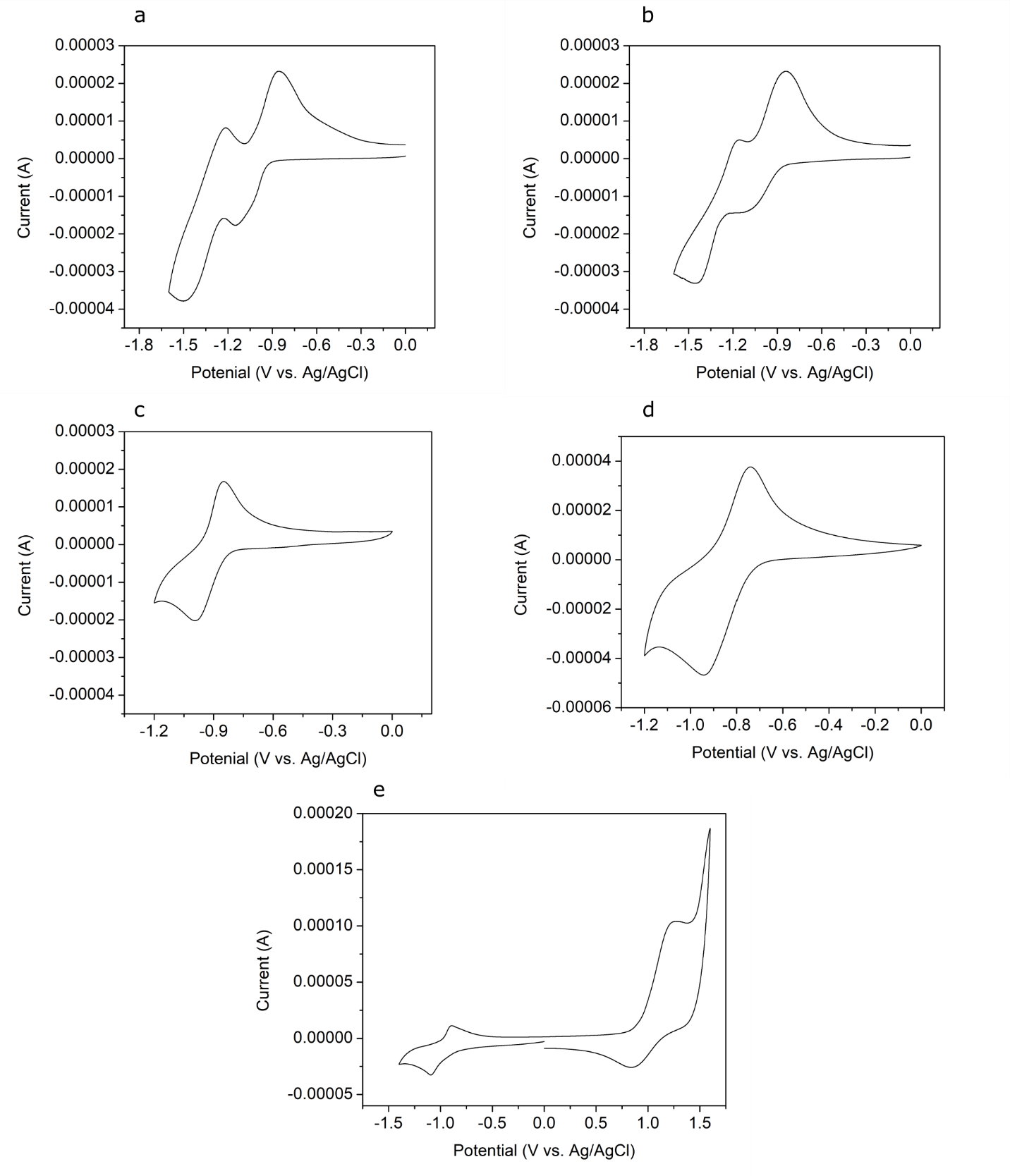


**Figure S1.** Cyclic voltammograms of each polymer. a. N2200 b. F-N2200 c. NDI-20-T d. NDIO-20-T e. PIBDFBT-37


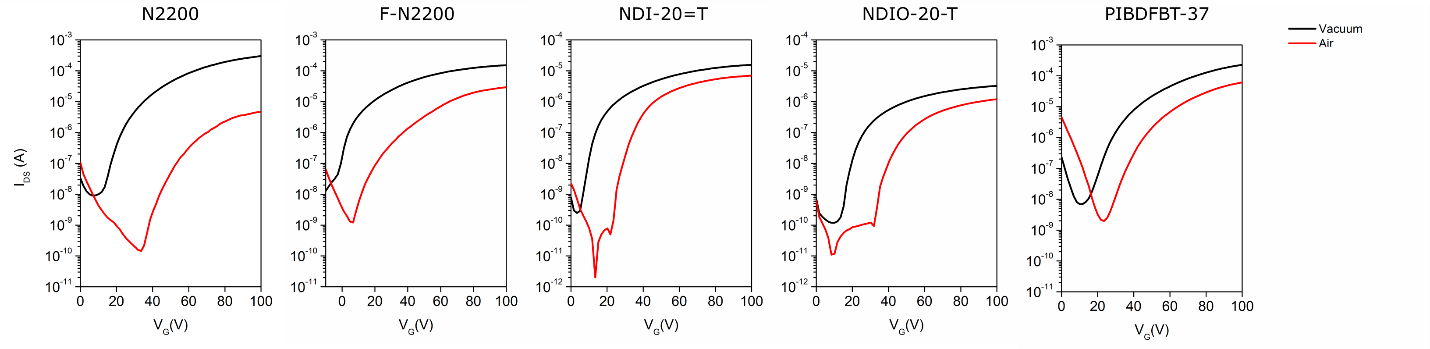


**Figure S2**. Transfer curves of each polymer at 30°C in air and vacuum.


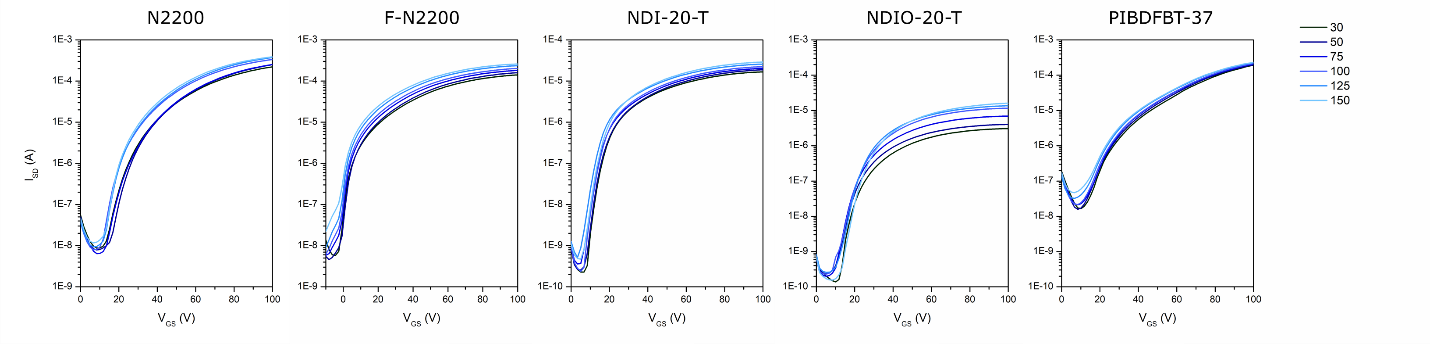


**Figure S3.** Transfer curves of each polymer from 30°C to 150°C in vacuum.


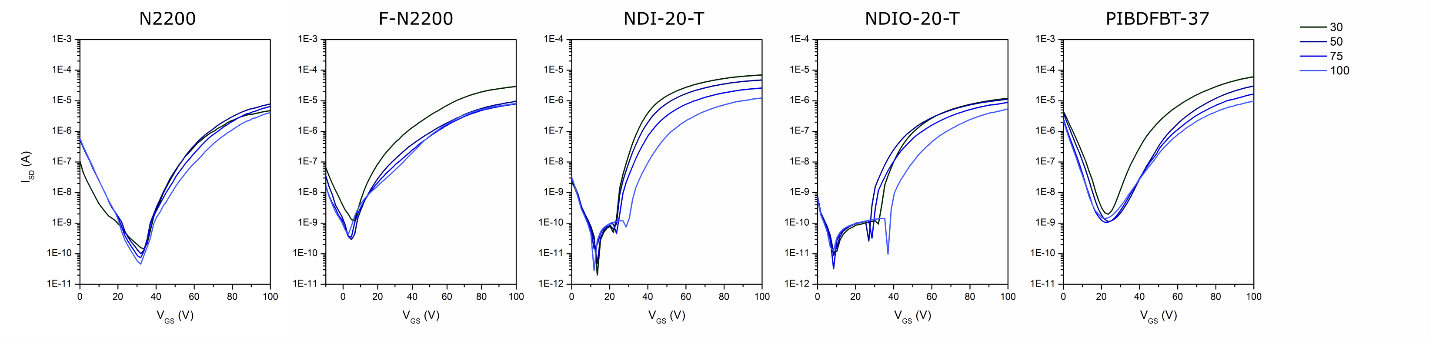


**Figure S4.** Transfer curves of each polymer from 30°C to 100°C in air.
